# Statin use is associated with improved survival in ovarian cancer: A retrospective population-based study

**DOI:** 10.1371/journal.pone.0189233

**Published:** 2017-12-19

**Authors:** Alexandra Couttenier, Olivia Lacroix, Evelien Vaes, Chris R. Cardwell, Harlinde De Schutter, Annie Robert

**Affiliations:** 1 Pôle d’Epidémiologie et Biostatistique, Institut de Recherche Expérimentale et Clinique, Université catholique de Louvain, Brussels, Belgium; 2 Research Department, Belgian Cancer Registry, Brussels, Belgium; 3 Centre for Public Health, Queen’s University Belfast, Belfast, Northern Ireland; Zhejiang University School of Medicine, CHINA

## Abstract

**Background:**

Preclinical in vitro and in vivo studies suggest that statins could exhibit anticancer properties in ovarian cancer. Similar effects have also been reported in observational studies but their results remain inconsistent and could be impaired by methodological limitations. This study aimed to investigate whether statin use is associated with improved survival in ovarian cancer patients at the Belgian population-level.

**Methods:**

All patients with invasive epithelial ovarian cancer diagnosed between 2004 and 2012 were identified from the Belgian Cancer Registry. Vital statuses were obtained from the Crossroads Bank for Social Security and ovarian cancer-specific deaths were identified from death certificates provided by regional administrations. Information on cancer treatments and statin use were retrieved from health insurance databases. Statin use was modelled as a time-varying covariate in Cox regression models to calculate adjusted hazards ratios (HR) and 95% confidence intervals (95%CI) for the association between postdiagnostic exposure to statins and overall- or ovarian cancer-specific mortality within three years after diagnosis. Adjustments were made for age at diagnosis, year of diagnosis, comorbidities, cancer stage, and cancer treatments.

**Results:**

A total of 5,416 patients with epithelial ovarian cancer met the inclusion criteria. Of these 1,255 (23%) had at least one statin prescription within three years after diagnosis. Postdiagnostic use of statins was associated with a reduced risk of overall mortality (adjusted HR = 0.81, 95%CI:0.72–0.90, p<0.001). In analyses by statin type, this association was only significant for simvastatin (adjusted HR = 0.86, 95%CI:0.74–0.99, p = 0.05) or rosuvastatin (adjusted HR = 0.71, 95%CI:0.55–0.92, p = 0.01). In subgroup analyses by statin prediagnostic use, the protective association for postdiagnostic statin use was only observed in patients who were also using statins before diagnosis (adjusted HR = 0.73, 95%CI:0.64–0.83, p<0.001). Similar results were observed for ovarian cancer-specific mortality.

**Conclusion:**

In this large nation-wide cohort of ovarian cancer patients postdiagnostic use of statins was associated with improved survival.

## Introduction

Statins are the most commonly used drugs to lower serum low-density lipoprotein (LDL) cholesterol levels. These drugs are prescribed among hypercholesterolemic patients in order to prevent cardiovascular diseases. [1–4] Statins are inhibitors of 3-Hydroxy-3-Methyl-Glutaryl-Coenzyme A Reductase enzyme (HMG-CoAR), the enzyme that catalyses the conversion of HMG-CoA to mevalonic acid. [4, 5]

By inhibiting this first and rate-limiting step in cholesterol biosynthesis, they may also provide an anticancer effect through two different ways. Firstly, by lowering cholesterol levels, tumour growth is counteracted as the proliferation of cancer cells requires cholesterol. [4] Secondly, statins prevent the activation of several oncogenic proteins such as Ras, Rho, Rac and Rab. Indeed, these oncoproteins need to be activated by isoprenoids which are intermediates of the HMG-CoA metabolic pathway. [4, 6]

Anticancer actions of statins would be interesting as new treatment perspectives in ovarian cancer (OC) which has a relatively poor prognosis with a 5-year survival rate around 40%. [7–9]

Several studies of human OC cell lines and animal ovarian cancer tumours models suggest that statins may have anticancer properties including induction of apoptosis and an inhibition of angiogenesis, tumour proliferation, invasion and metastasis. [10–14]

Following these findings, three clinical series were conducted which suggested that OC patients who used statins had an improved survival compared to nonusers. [15–17] However, the results of these studies are debated and could reflect limitations such as small sample sizes, absence of detailed information on statin use, and a lack of control for potential time-related biases. So far, at the population level, there is no study addressing the hypothesis of an increased survival in OC patients who use statins.

The present study was conducted in order to assess whether there is an association between statin use and survival in patient diagnosed with OC at Belgian population-level. We used population-based data from the Belgian Cancer Registry (BCR) linked to mortality data and health administrative databases. We compared survival between OC patients who used statins and patients who did not use statins. These data gathered at BCR form an ideal setting to exert pharmacological studies on the effect of chronic exposure to non-oncological medication on cancer outcome, as in the current study on statins and OC survival.

## Materials and methods

### Study design and data sources

This population-based cancer-registry study was conducted in the BCR. The BCR routinely collects information regarding new cancer diagnoses in Belgium including patient characteristics (such as age at diagnosis, region of residence) and tumour characteristics (such as date of diagnosis, cancer topography and morphology) on a national level since the incidence year 2004. In Belgium, the specific Health Law of December 13th 2006 [18] has made cancer registration compulsory for the pathological anatomy laboratories and for the oncological care programs. Moreover, the reimbursement of multidisciplinary oncological consultation for each reported case of cancer acts as an incentive for the cancer registration. This provides high quality data and high completeness of BCR database, which is estimated to be more than 95% complete using cross-validation techniques. [19] The remaining incompleteness is most likely due to elderly patients with a very poor prognosis at diagnosis and outpatients with a clinical diagnosis only. Based on the aforementioned law, the BCR has also the legal authorization to use the National Social Security Number (NSSN) as a unique patient identifier. This NSSN allows the BCR to deterministically link its data with data from the Crossroads Bank for Social Security (CBSS) to retrieve information on vital status. The NSSN can also be used to deterministically link the BCR data with reimbursement data from the health insurance companies, provided by the Intermutualistic Agency (IMA). As health insurance is mandatory in Belgium, administrative, medical claims data from IMA are available for almost the entire Belgian population. The IMA database contains information regarding all reimbursed medications and medical acts. Since statins are reimbursed drugs that are only available on prescription in Belgium, all dispensed statin prescriptions can be identified within IMA data. In addition, for more than 90% of the BCR population, [19] death certificates provided by the regional administrations can be used to retrieve causes of death using a linkage based on several variables (such as date of birth, date of death, sex, residence at time of death).

The linkage of the BCR-, the IMA-, the CBSS- and the regional administrations’ databases thus provides information on all patients diagnosed with cancers in Belgium, on their vital status, their date and cause of death, the cancer treatments they received, and their medication use.

Specific ethical approvals were not required because the analyses were conducted within the legal framework of the BCR, using only anonymized data and without any access to private information on individual patients.

### Study population

All patients with an OC (ICD-10 code C56) diagnosed between 2004 and 2012 were identified from the BCR.

Patients with borderline malignant tumours or with non-epithelial cancers were excluded. Patients with another invasive cancer diagnosis (apart from non-melanoma skin cancer) prior to their OC diagnosis were also excluded. Additionally, patients who died within six months after OC diagnosis were excluded as these patients were mainly diagnosed in advance stage disease. It seems unlikely that short term postdiagnostic medication usage could influence their survival. Therefore, the beginning of the follow-up time was set to 6 months after diagnosis. The other exclusion criteria were related to missing data or absence of follow-up data, thus, patients were also excluded if they did not have an available NSSN or available IMA data, if they died on the day of diagnosis or if they were lost to follow-up at diagnosis. Exclusions based on these criteria are expected to be rare in our study population because in the general BCR database, only 1.5% and 3.4% of patients don’t have a NSSN or IMA data available, only 0.16% of patients died on the day of diagnosis and 1.2% of patients are lost to follow-up at diagnosis. [20]

Patients were followed from six months after diagnosis to a maximum of 3 years after their OC diagnosis (or to the end of follow-up on July 1st, 2015).

### Exposition variables and covariates

Use of statins was identified from IMA data using the Anatomical Therapeutic Chemical (ATC) codes ‘C10AA’.

To avoid immortal time bias, [21] statin use was modelled as a time varying covariate, i.e. patients were initially considered as nonusers and then became users after their first statin prescription. As recommended in studies of medication use and cancer survival, [22] a lag period of 6 months was applied to exclude prescriptions that fall within the 6 months period prior to death because these may be affected by palliative treatments.

Statins were also examined by each statin type and by subgroups of statins according to solubility type, i.e. lipophilic agents (simvastatin and fluvastatin) and hydrophilic agents (atorvastatin, pravastatin and rosuvastatin).

Statin exposure was further investigated in a dose-response analysis by counting the total daily defined doses (DDD) of statins that the patient received during the follow-up. After their first statin prescription, patients were considered as light users and they became heavy users as soon as they cumulated at least 365/2 DDDs.

The available covariates were age at diagnosis, year of cancer diagnosis, histological grade, combined stage (according to the TNM Classification of Malignant Tumours, 7th edition), cancer histologic subtype, and cancer treatments (including surgery, chemotherapy, and radiotherapy in the 9 months after diagnosis). Cardiovascular comorbidities and diabetes were estimated using a previously described methodology based on specific drugs prescriptions in the year before the diagnosis. [23]

### Statistical analysis

Descriptive statistics were used to characterize patients at the time of diagnosis. These analyses were conducted within subgroups of statin users and nonusers in order to compare characteristics in these two groups. Patient- and tumor characteristics in each group are reported as frequencies and percentages. Age was the only variable considered both in categories and as a continuous variable.

Time-dependent Cox regression models were used to calculate hazard ratios (HRs) and 95% confidence intervals (95% CIs) for postdiagnostic statin users compared to nonusers and risk of overall mortality within 3 years after diagnosis. Analyses were adjusted for the following potential confounders: age in categories (≤ 49 years, 50–74 years, ≥ 75 years), year of diagnosis (in 3-year bands: 2004 to 2006, 2007 to 2009 and 2010 to 2012), cancer stage (I, II, III, IV), cancer treatments (neoadjuvant chemotherapy, adjuvant chemotherapy, chemotherapy only, surgery only and no treatment), cardiovascular comorbidities (yes or no) and diabetes (yes or no).

Subgroup analyses were conducted by age categories, year of diagnosis, cancer stage, histologic subtype, cancer treatment and prediagnostic statin use. Tests for interactions were performed using interaction terms within Cox regression models.

A sensitivity analysis that controls for immortal time bias without requiring time varying covariate was conducted. This simplified analysis used Cox regression to compare statin users to statin nonusers in the first 6 months after diagnosis.

After the exclusion of deceased patients having missing death certificates (n = 521, 10%), analyses were also performed for ovarian cancer specific-mortality (S1 and S2 Tables and S1 Fig).

The statistical significance level was set to P<0.05. All analyses were performed using the statistical software SAS Enterprise Guide 5.1.

## Results

### Patient characteristics

**Fig 1:**
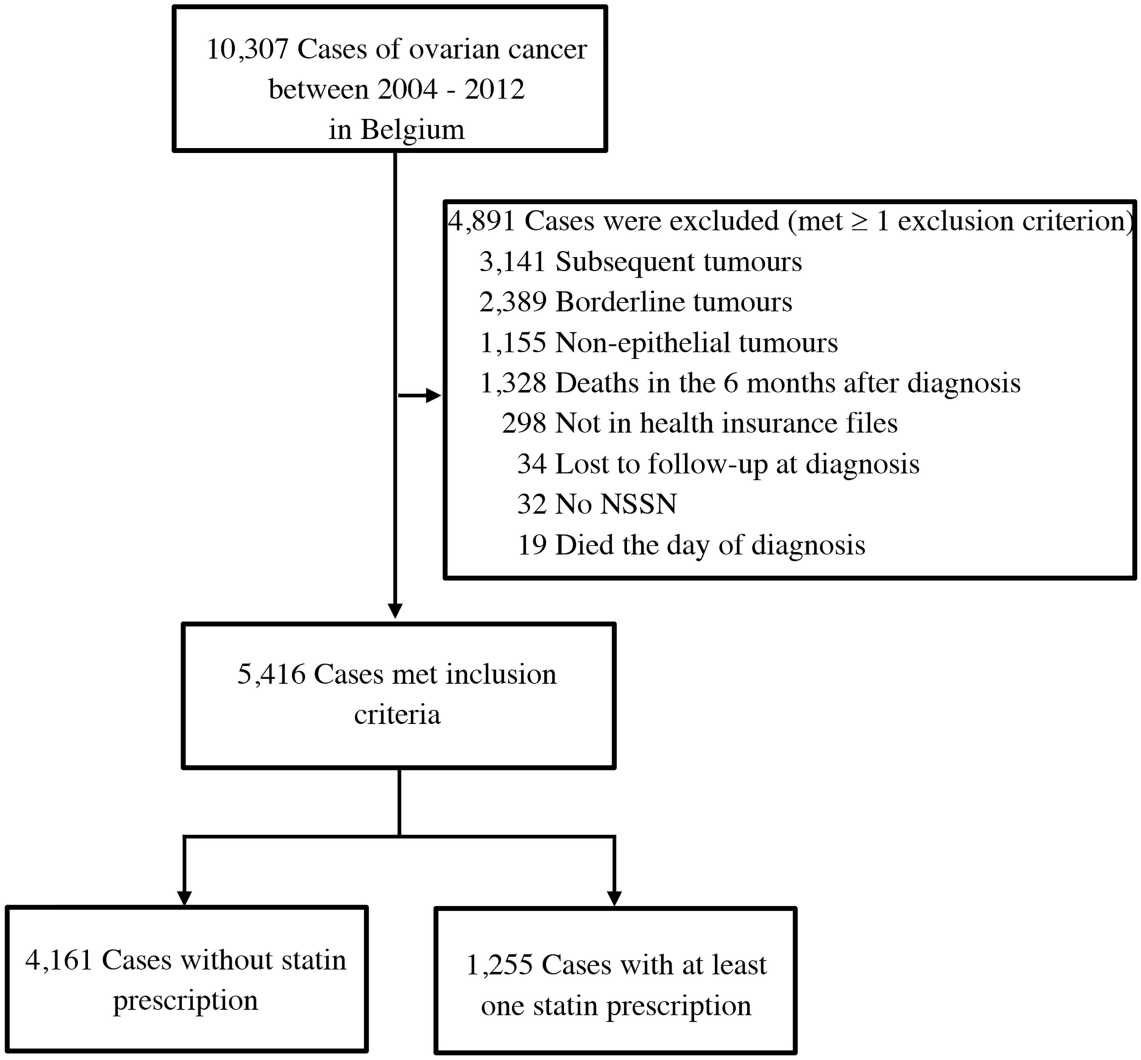
Flowchart of patients. NSSN stands for the National Social Security Number in Belgium.

A flowchart of the selection of patients for the study is shown in Fig 1. A total of 10,307 patients diagnosed with OC between 2004 and 2012 were identified from the BCR. Of these, 4,891 patients were excluded because they met at least one exclusion criterion. Exclusions based on tumor characteristics comprised 2,389 patients with borderline tumors, 1,155 patients with non-epithelial tumors and 710 patients with another invasive cancer diagnosis prior to their OC diagnosis. In total 298 patients could not be linked to the health insurance databases and 32 patients did not have a NSSN. Furthermore, exclusion criteria regarding follow-up were met in 1,328 patients who died within the 6 months following the diagnosis, as well as 34 patients who were lost to follow-up at diagnosis, and 19 patients who died on their date of diagnosis.

Among the remaining 5,416 patients diagnosed with OC, almost one quarter (1,255 patients, 23%) received at least one statin prescription in the 3 years after their cancer diagnosis. Patient characteristics for statin users and nonusers are shown in Table 1. On average, statin users were 6 years older at diagnosis compared to nonusers. Statin users were also more likely to be diagnosed more recently compared to nonusers. As expected from a clinical point of view, the prevalence of comorbidities was higher in statin users (15% with diabetes and 35% with cardiovascular diseases in users compared to 6% and 30% in nonusers). Tumour characteristics such as stage, grade, and morphology were generally similar between the two groups. The majority of patients had advanced stage (45% at stage III or IV), advanced grade disease (45% at grade 3 or 4), and had a serous histology (73%). Receipt of cancer treatments was also similar between the two groups, with a majority of patients undergoing both surgery and chemotherapy (62%).

**Table 1:** Characteristics of ovarian cancer patients by statin use after diagnosis.

	Statin use after diagnosis	
Characteristic	User (n = 1,255)	Nonuser (n = 4,161)
Age at diagnosis − yrs*	68±10	62±14
Categories of age at diagnosis − n (%)		
≤ 49 years	55 (4)	716 (17)
50–74 years	846 (68)	2570 (62)
≥ 75 years	354 (28)	875 (21)
Year of diagnosis − n (%)		
2004–2006	367 (29)	1539 (37)
2007–2009	441 (35)	1375 (33)
2010–2012	447 (36)	1247 (30)
Cancer stage − n (%)		
I	249 (20)	739 (18)
II	75 (7)	238 (6)
III	340 (26)	1168 (28)
IV	200 (16)	715 (17)
Unknown	391 (31)	1300 (31)
Not assessed	0	1
Grade of differentiation − n (%)		
1	123 (11)	411 (12)
2	215 (20)	696 (19)
3	444 (41)	1479 (41)
4	32 (4)	145 (4)
Unknown	261 (24)	864 (24)
Not assessed	180	566
Histologic Subtype − n (%)		
Serous	762 (61)	2499 (60)
Mucinous	93 (7)	404 (10)
Endometrioid	124 (10)	321 (8)
Clear cell	54 (4)	186 (4)
Others or unspecified	222 (18)	751 (18)
Cancer treatment (within 9 months) − n (%)		
None (+ RT only)	42 (3)	200 (5)
Surgery only	247 (20)	730 (18)
Chemotherapy only	183 (14)	645 (15)
Neoadjuvant chemotherapy	300 (24)	959 (23)
Adjuvant chemotherapy	483 (39)	1627 (39)
Comorbidities before cancer diagnosis − n (%)		
Diabetes	192 (15)	259 (6)
Cardiovascular diseases	709 (56)	1292 (30)

*mean ± standard deviation. RT denotes radiotherapy.

### Association between postdiagnostic statin use and all-cause mortality

Results from Cox regression models for the association between postdiagnostic statin use and overall mortality are shown in Table 2. After adjustment for potential confounders, statin users after diagnosis had a 19% reduction in mortality (adjusted HR, 0.81; 95% CI, 0.72–0.90; P<0.001) when compared with nonusers.

**Table 2:** Association between statin use after diagnosis and overall mortality in patients with ovarian cancer.*

				Unadjusted		Adjusted^a^	
Medication usage after diagnosis	Patients n	Deaths n (%)	Person-Years	HR [95%CI]	P	HR [95%CI]	P
Statin nonuser^b^	4161	1619 (39)	10679.2	Referent		Referent	
Statin user^b^	1255	420 (33)	2678.9	0.98 [0.88;1.09]	0.69	0.81 [0.72;0.90]	<.001
<365 /2 DDDs^c^	375	190 (51)	1634.6	0.99 [0.86;1.14]	0.89	0.80 [0.70;0.93]	0.003
≥365 /2 DDDs^c^	880	230 (26)	1245.3	1.05 [0.92;1.21]	0.46	0.87 [0.76;1.01]	0.06
Lipophilic nonuser^d^	4789	1828 (38)	12039.7	Referent		Referent	
Lipophilic user^d^	627	211 (34)	1318.4	1.00 [0.87;1.16]	0.97	0.87 [0.75;1.01]	0.06
Simvastatin nonuser	4806	1835 (38)	12072.9	Referent		Referent	
Simvastatin user	610	204 (33)	1285.2	0.99 [0.86;1.15]	0.92	0.86 [0.74;0.99]	0.05
Fluvastatin nonuser	5399	2032 (38)	13324.9	Referent		Referent	
Fluvastatin user	17	7 (41)	33.2	1.35 [0.65;2.84]	0.42	1.26 [0.60;2.66]	0.54
Hydrophilic nonuser^e^	4788	1830 (38)	11997.6	Referent		Referent	
Hydrophilic user^e^	628	209 (33)	1360.5	0.96 [0.83;1.11]	0.58	0.81 [0.70;0.93]	0.003
Pravastatin nonuser	5303	1998 (38)	13111.3	Referent		Referent	
Pravastatin user	113	41 (36)	246.8	1.05 [0.77;1.43]	0.77	0.87 [0.64;1.18]	0.37
Atorvastatin nonuser	5111	1930 (38)	12714.3	Referent		Referent	
Atorvastatin user	305	109 (36)	643.8	1.06 [0.88;1.29]	0.53	0.88 [0.72;1.06]	0.18
Rosuvastatin nonuser	5206	1980 (38)	12888.2	Referent		Referent	
Rosuvastatin user	210	59 (28)	469.9	0.78 [0.60;1.01]	0.06	0.71 [0.55;0.92]	0.01

* CI denotes confidence interval and DDD denotes Defined Daily Dose.

^*a*^ Adjusted model contains age in categories (≤49 years, 50–74 years, ≥75 years), year of diagnosis (in 3-years bands), stage, cancer treatment within the 9 months (none, surgery only, chemotherapy only, neoadjuvant and adjuvant chemotherapy), comorbidities (diabetes and cardiovascular diseases).

^*b*^ Statin use defined as at least one statin prescription after diagnosis.

^*c*^ Survival of users with less than 365/2 DDDs or with at least 365/2 DDDs are compared with survival of nonusers.

^*d*^ Lipophilic statins include simvastatin and fluvastatin.

^*e*^ Hydrophilic statins include pravastatin, atorvastatin and rosuvastatin.

There was no evidence of a dose-response association when statin use was investigated by increasing number of DDDs. Compared with statin nonusers, the reduction in risk of death was similar in patients with less than 365/2 DDDs (adjusted HR, 0.80; 95% CI, 0.70–0.93; P = 0.003) and in those with at least 365/2 DDDs (adjusted HR, 0.87; 95% CI, 0.76–1.01; P = 0.06).

Overall, the mortality reduction in statin users compared to nonusers was more marked for hydrophilic agents (adjusted HR, 0.81; 95% CI 0.70–0.93; P = 0.003) than for lipophilic agents (adjusted HR, 0.87; 95% CI 0.75–1.01; P = 0.06). In separate analyses of statin type, only the hydrophilic rosuvastatin and the lipophilic simvastatin appeared to have significant protective associations (adjusted HR, 0.76; 95% CI, 0.55–0.92; P = 0.01 and adjusted HR, 0.86; 95% CI, 0.74–0.99, P = 0.05, respectively). Similar associations were observed for cancer-specific mortality (S1 Table).

### Secondary and sensitivity analyses

**Fig 2:**
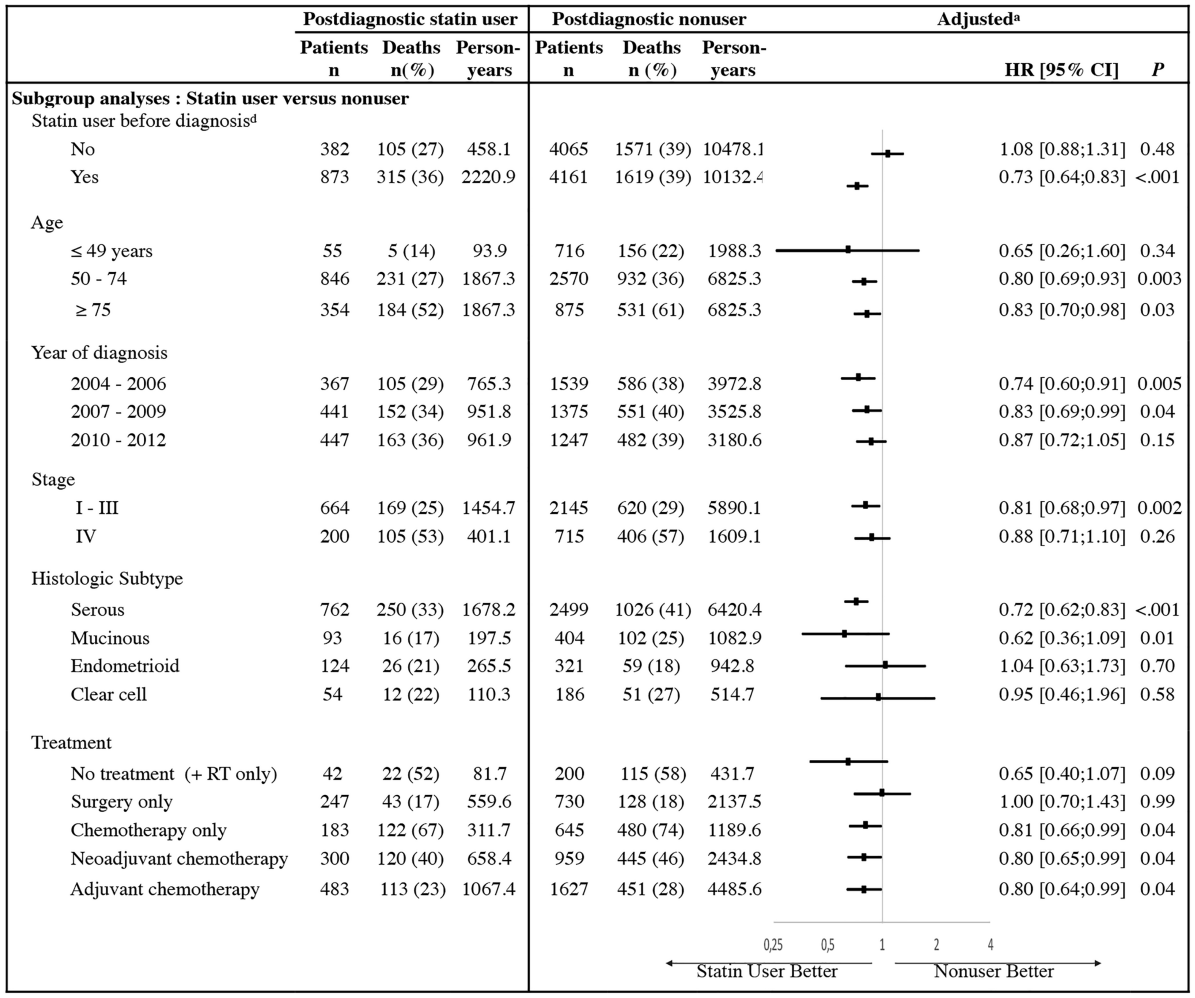
Subgroup analyses of the association between statin use after diagnosis and overall mortality in patients with ovarian cancer.* The study population was divided into subgroups according to several categorical covariates. A horizontal line on the figure represents one subgroup and it is divided between postdiagnostic statin users and nonusers (with the size, the number of deaths (+%), and the number of person-years). For each subgroup (ie. line), the survival of postdiagnostic statin users and nonusers were compared and the obtained estimations were graphically represented and reported as HR (CI95%) with corresponding P-values. * CI denotes confidence interval. ^*a*^ Adjusted model contains age in categories (≤49 years, 50–74 years, ≥75 years), year of diagnosis (in 3-years bands), stage, cancer treatment within the 9 months (none, surgery only, chemotherapy only, neoadjuvant and adjuvant chemotherapy), comorbidities (diabetes and cardiovascular diseases). ^*b*^ Statin use before diagnosis was defined as at least one statin prescription in the year prior to the diagnosis.

Subgroup analyses by age, year of diagnosis, cancer stage, histologic subtype, cancer treatment and prediagnostic statin use are shown in Fig 2. Interaction tests that investigated the potential change in the association between postdiagnostic statin use and survival within different subgroups of covariates were carried out. This showed that only stratification by prediagnostic statin use modified the association between postdiagnostic statin use and survival (P_*interaction*_<0.0001). Indeed, the reduction in mortality associated with postdiagnostic statin use was observed only in patients that had also used statins prior to diagnosis (adjusted HR, 0.73; 95% CI, 0.64–0.83; P<0.001) and not in patients with no prediagnostic use (adjusted HR, 1.08; 95% CI, 0.88–1.31; P = 0.48). In other subgroup analyses, there were no marked differences in the associations between statin postdiagnostic use and survival statified by age (P_*interaction*_ = 0.77), year of diagnosis (P_*interaction*_ = 0.31), stage (P_*interaction*_ = 0.71), histology (P_*interaction*_ = 0.19) or treatments (P_*interaction*_ = 0.55). Similar results were observed for ovarian cancer specific-mortality (S1 Fig).

Results of the sensitivity analysis are displayed in Table 3. In this simplified analysis, statin use in the six months after diagnosis was associated with a non-statistically significant mortality reduction in OC patients (adjusted HR, 0.90; 95% CI, 0.81–1.00; P = 0.06). A similar finding was observed for ovarian cancer-specific survival (S2 Table).

**Table 3:** Sensitivity analysis of association between statin use and overall mortality in patients with ovarian cancer.

				Unadjusted		Adjusted^a^	
6 months postdiagnostic use	Patients n	Deaths n (%)	Person-Years	HR [95%CI]	P	HR [95%CI]	P
Statin nonuser^b^	4289	1577(37)	10623.6	Referent		Referent	
Statin user^b^	1127	462(41)	2734.5	1.14 [1.03;1.27]	0.01	0.90 [0.81;1.00]	0.06

^*a*^ Adjusted model contains age in categories (≤49 years, 50–74 years, ≥75 years), year of diagnosis (in 3-years bands), stage, cancer treatment within the 9 months (none, surgery only, chemotherapy only, neoadjuvant and adjuvant chemotherapy), comorbidities (diabetes and cardiovascular diseases).

^*b*^ Statin use defined as at least one statin prescription in the first 6 months after diagnosis

## Discussion

In this large population-based cohort, OC patients who used statins after diagnosis had a significant 19% reduction in overall mortality compared to statin nonusers (adjusted HR, 0.81; 95% CI, 0.72–0.90; P<0.001.) A similar association was observed between postdiagnostic statin use and ovarian cancer-specific mortality, with a significant 18% reduction in ovarian cancer specific-mortality (adjusted HR, 0.82; 95% CI, 072–0.93; P = 0.002).

Our results support the findings of three earlier clinical series of statin use and OC outcomes after diagnosis. [15–17] However, the protective association for statins observed in our study was less marked. Elmore et al. [15] observed that OC patients using statins at the time of primary surgery had a 55% reduction in mortality (adjusted HR = 0.45; 95% CI, 0.23–0.88; P = 0.02). This single center study, based on a small sample (17 users), only investigated preoperative statin use and lacked information on statin dose and duration. Lavie et al. [16] reported a similar reduction of mortality in OC patients who used postiagnostic statins (age adjusted HR = 0.47; 95% CI, 0.26–0.85). This study also enrolled a small number of patients (16 users) and didn’t control for potential time-related biases. Habis et al. [17] also observed a substantial protective association between statin use and OC survival but only in a secondary analysis in a small subgroup of patients (14 users) with non-serous-papillary subtypes of OC (adjusted HR = 0.23, P = 0.04). However, no interaction test was performed to assess the heterogeneity of statins effect in the subgroup analysis by tumour histology.

As such, the latter study contradicts the findings from the other studies that observed a benefit of statin usage in their entire OC cohort containing more than 90% of serous tumors. After our main analyses that were based on all epithelial OC patients, further subgroup analyses were conducted to assess wether statin effects were heterogeneous in histologic subtypes. In these secondary analyses, we did not identify any significant modification of the association between statin use and survival in different subgroups of epithelial OC histologies (P_*interaction*_ = 0.19).

To our knowledge this study is the first to investigate the association between OC survival and statin use by statin solubility or by statin type. Overall, both lipophilic and hydrophilic statins showed protective associations for mortality. However, in more detailed analyses, only simvastatin and rosuvastatin reached a significantly protective effect (adjusted HR, 0.86; 95% CI, 0.74–0.99, P = 0.05 and adjusted HR, 0.71; 95% CI, 0.55–0.92, P = 0.01, respectively). These results are corroborated by preclinical studies that report varying tumor-suppressive effects for different statin types in various cancers. These preclinical studies suggest that under in vitro conditions, simvastatin exerts the largest tumor-suppressive effects. While based on in vivo results, rosuvastatin and fluvastatin were the most potent compounds in animal models. [24, 25]

No dose-response effects were observed in our analyses, which suggests that any statin dose may be sufficient to reduce mortality risk among patients with OC. Although the previous studies in OC patients didn’t investigate the influence of statin dose, our results are consistent with findings in other cancer sites. For instance, several studies examining statin use and breast cancer survival have not observed any dose-response associations. [26–28]

In secondary analyses, we investigated the influence of postdiagnostic statin use by prediagnostic statin use in the year prior to diagnosis and found that only patients who were using statins both before and after diagnosis of OC experienced a significant reduction in mortality compared to nonusers. This observation contradicts the findings from the study of Lavie et al., [16] which is the only previous study to investigate the influence of prediagnositic use of statins on OC survival. In their study, the authors observed that patients who used statins only after diagnosis had a survival advantage compared to nonusers, and to women who used statins only before diagnosis, but also, to women who used statins prior to diagnosis and continued using statins after diagnosis. However, it was a small study and the authors did not control for potential time-related bias, thus, the patients that were classified as statin users only after diagnosis, will spuriously appear to have a better survival compared to other analysis groups. [21] Population-based studies in breast cancer have reported comparable results to ours with lower breast cancer mortality in sub-analyses. [27–31] It is unclear what the general clinical impact of prediagnostic statin use could be since it is difficult to envisage any interventions in the prediagnostic period. However, our finding of a protective association for both prediagnostic use and postdiagnostic use suggests that OC patients taking statins before the diagnosis should continue using statins after diagnosis. Moreover, it may also suggest that women with high risk for OC could possibly start a “preventive” statin therapy in order to have a survival benefit would they experience this disease. On the other hand, as the association between postdiagnostic statin use and survival was not significant for OC patients who did not take statins before diagnosis, we cannot suggest whether starting a statin therapy in these patients could still be beneficial.

Our study was the first to conduct analyses stratified by treatments in OC patients. Studies based on ovarian cancer cells lines found conflicting evidence regarding the combination of statins and chemotherapies. [12, 32] In fact, while some in vitro studies suggested a synergic combination, others expected an antagonist action. In our analyses, there was no proof that chemotherapies modify the effect of statins since there were no marked differences in associations by treatment subgroups.

Similarly, in the other subgroup analyses, there were no marked differences in the associations by year of incidence, age, or by stage.

Unfortunately, and although we adjusted for potential confounders, we cannot rule out confounding effects in the observed associations particularly for unrecorded covariates (such as BMI or other comorbidities).

Nevertheless, this study shows several strengths. This is the largest study yet to investigate the association between the use of statins and survival in ovarian cancer patients. Our study size ensures a good statistical power. Results in all-cause mortality analyses and OC specific-mortality analyses (S1 and S2 Tables and S1 Fig) were very similar. Linkages to mortality registries and death certificates ensure a robust identification of deaths and ovarian cancer specific deaths (although some misclassification remains possible). The use of health insurance data avoided any recall bias and provided precise information regarding statin use including the timing of medication dispensing, dosages, and statins types. These detailed data permitted exploration of temporal relationships between statin use and mortality and also allowed analyses by statin types, doses and solubility. In addition, availability of clinical factors such as cancer stage, histology, and cancer treatments facilitated subgroup analyses.

To conclude, this large nationwide study showed some evidence of a protective effect of statin use on ovarian cancer-specific and all-cause mortality. This mortality reduction was not significantly modified by age, year of diagnosis, cancer histology, stage, or cancer treatment. Simvastatin and rosuvastatin in particular appeared to have the strongest protective associations. The protective association for postdiagnostic statin use was not dose-dependent but seemed to be more beneficial in patients already using statins before their diagnosis. Since it remains difficult to envisage statin interventions during the prediagnostic period, a clinical implication for the general population is not straightforward but our research suggests that women at high risk for developing OC could possibly benefit from preventive statin use. Further other population-based studies are required to confirm our findings.

## Supporting information

S1 Table Association between statin use after diagnosis and cancer-specific mortality in patients with ovarian cancer.* * CI denotes confidence interval and DDD denotes Defined Daily Dose.
^*a*^ Adjusted model contains age in categories (≤49 years, 50–74 years, ≥75 years), year of diagnosis (in 3-years bands), stage, cancer treatment within the 9 months (none, surgery only, chemotherapy only, neoadjuvant and adjuvant chemotherapy), comorbidities (diabetes and cardiovascular diseases).
^*b*^ Statin use defined as at least one statin prescription after diagnosis.
^*c*^ Survival of users with less than 365/2 DDDs or with at least 365/2 DDDs are compared with survival of nonusers.
^*d*^ Lipophilic statins include simvastatin and fluvastatin.
^*e*^ Hydrophilic statins include pravastatin, atorvastatin and rosuvastatin.
(PDF)

S2 Table Sensitivity analysis of association between statin use and cancer-specific mortality in patients with ovarian cancer. ^*a*^ Adjusted model contains age in categories (≤49 years, 50–74 years, ≥75 years), year of diagnosis (in 3-years bands), stage, cancer treatment within the 9 months (none, surgery only, chemotherapy only, neoadjuvant and adjuvant chemotherapy), comorbidities (diabetes and cardiovascular diseases).
^*b*^ Statin use defined as at least one statin prescription in the first 6 months after diagnosis.
(PDF)

S1 Fig Subgroup analyses of the association between statin use after diagnosis and cancer specific-mortality in patients with ovarian cancer.* * CI denotes confidence interval. ^*a*^ Adjusted model contains age in categories (≤49 years, 50–74 years, ≥75 years), year of diagnosis (in 3-years bands), stage, cancer treatment within the 9 months (none, surgery only, chemotherapy only, neoadjuvant and adjuvant chemotherapy), comorbidities (diabetes and cardiovascular diseases). ^*b*^ Statin use before diagnosis was defined as at least one statin prescription in the year prior to the diagnosis.
(TIF)
