# Supplementary material for: Statin use is associated with improved survival in ovarian cancer: A retrospective population-based study
Source: PLoS One. 2017 Dec 19;12(12):e0189233. doi: 10.1371/journal.pone.0189233 (PMC5736195; doi:10.1371/journal.pone.0189233)
Supplement: S1 Table — * CI denotes confidence interval and DDD denotes Defined Daily Dose. a Adjusted model contains age in categories (≤49 years, 50–74 years, ≥75 years), year of diagnosis (in 3-years bands), stage, cancer treatment within the 9 months (none, surgery only, chemotherapy only, neoadjuvant and adjuvant chemotherapy), comorbidities (diabetes and cardiovascular diseases). b Statin use defined as at least one statin prescription after diagnosis. c Survival of users with less than 365/2 DDDs or with at least 365/2 DDDs are compared with survival of nonusers. d Lipophilic statins include simvastatin and fluvastatin. e Hydrophilic statins include pravastatin, atorvastatin and rosuvastatin. (PDF) [file pone.0189233.s001.pdf]

Table S1: **Association between statin use after diagnosis and cancer-specific mortality in patients with ovarian cancer.***

				Unadjusted		Adjusted[a]	
Medication usage after diagnosis	Patients n	Deaths n (%)	Person-Years	HR [95%CI]	P	HR [95%CI]	P
Statin nonuser[b]	3777	1250 (33)	9586.9	Referent		Referent	
Statin user[b]	1118	317 (28)	2355.9	0.98 [0.87;1.11]	0.78	0.82 [0.72;0.93]	0.002
$<$365 /2 DDDs[c]	348	154 (44)	1458.2	1.02 [0.87;1.19]	0.85	0.83 [0.71;0.97]	0.02
$\geq$365 /2 DDDs[c]	770	163 (21)	1074.9	1.04 [0.88;1.22]	0.67	0.86 [0.73;1.02]	0.08
Lipophilic nonuser[d]	4335	1412 (33)	10783.1	Referent		Referent	
Lipophilic user[d]	560	155 (28)	1159.8	0.98 [0.83;1.15]	0.78	0.85 [0.72;1.00]	0.06
Simvastatin nonuser	4352	1419 (33)	10816.3	Referent		Referent	
Simvastatin user	543	148 (27)	1126.5	0.96 [0.81;1.14]	0.62	0.83 [0.70;0.99]	0.04
Fluvastatin nonuser	4878	1560 (32)	11909.6	Referent		Referent	
Fluvastatin user	17	7 (41)	33.2	1.58 [0.75;3.32]	0.23	1.43 [0.68;3.00]	0.35
Hydrophilic nonuser[e]	4337	1405 (32)	10746.7	Referent		Referent	
Hydrophilic user[e]	558	162 (29)	1196.2	0.99 [0.84;1.17]	0.92	0.84 [0.71;0.99]	0.03
Pravastatin nonuser	4794	1532 (32)	11727.4	Referent		Referent	
Pravastatin user	101	35 (35)	215.5	1.20 [0.86;1.67]	0.29	1.06 [0.76;1.48]	0.75
Atorvastatin nonuser	4620	1482 (32)	11368.9	Referent		Referent	
Atorvastatin user	275	85 (31)	574.0	1.09 [0.87;1.35]	0.46	0.87 [0.70;1.09]	0.22
Rosuvastatin nonuser	4713	1525 (32)	11536.2	Referent		Referent	
Rosuvastatin user	182	42 (23)	406.7	0.75 [0.55;1.02]	0.07	0.70 [0.51;0.95]	0.02

* CI denotes confidence interval and DDD denotes Defined Daily Dose.

[a] Adjusted model contains age in categories ($\leq$49 years, 50-74 years, $\geq$75 years), year of diagnosis (in 3-years bands), stage, cancer treatment within the 9 months (none, surgery only, chemotherapy only, neoadjuvant and adjuvant chemotherapy), comorbidities (diabetes and cardiovascular diseases).

[b] Statin use defined as at least one statin prescription after diagnosis.

[c] Survival of users with less than 365/2 DDDs or with at least 365/2 DDDs are compared with survival of nonusers.

[d] Lipophilic statins include simvastatin and fluvastatin.

[e] Hydrophilic statins include pravastatin, atorvastatin and rosuvastatin.
