# Supplementary material for: Statin use is associated with improved survival in ovarian cancer: A retrospective population-based study
Source: PLoS One. 2017 Dec 19;12(12):e0189233. doi: 10.1371/journal.pone.0189233 (PMC5736195; doi:10.1371/journal.pone.0189233)
Supplement: S2 Table — a Adjusted model contains age in categories (≤49 years, 50–74 years, ≥75 years), year of diagnosis (in 3-years bands), stage, cancer treatment within the 9 months (none, surgery only, chemotherapy only, neoadjuvant and adjuvant chemotherapy), comorbidities (diabetes and cardiovascular diseases). b Statin use defined as at least one statin prescription in the first 6 months after diagnosis. (PDF) [file pone.0189233.s002.pdf]

Table S1: **Sensitivity analysis of association between statin use and cancer-specific mortality in patients with ovarian cancer.**

				Unadjusted		Adjusted[a]	
6 months postdiagnostic use	Patients n	Deaths n (%)	Person-Years	HR [95%CI]	P	HR [95%CI]	P
Statin nonuser[b]	3890	1218 (31)	9536.9	Referent		Referent	
Statin user[b]	1005	349 (35)	2405.9	1.14 [1.01;1.28]	0.03	0.90 [0.80;1.02]	0.10

[a]Adjusted model contains age in categories ($\leq$49 years, 50-74 years, $\geq$75 years), year of diagnosis (in 3-years bands), stage, cancer treatment within the 9 months (none, surgery only, chemotherapy only, neoadjuvant and adjuvant chemotherapy), comorbidities (diabetes and cardiovascular diseases).
[b]Statin use defined as at least one statin prescription in the first 6 months after diagnosis
