# Supplementary figures and images for: Statin use is associated with improved survival in ovarian cancer: A retrospective population-based study
Source: PLoS One. 2017 Dec 19;12(12):e0189233. doi: 10.1371/journal.pone.0189233 (PMC5736195; doi:10.1371/journal.pone.0189233)

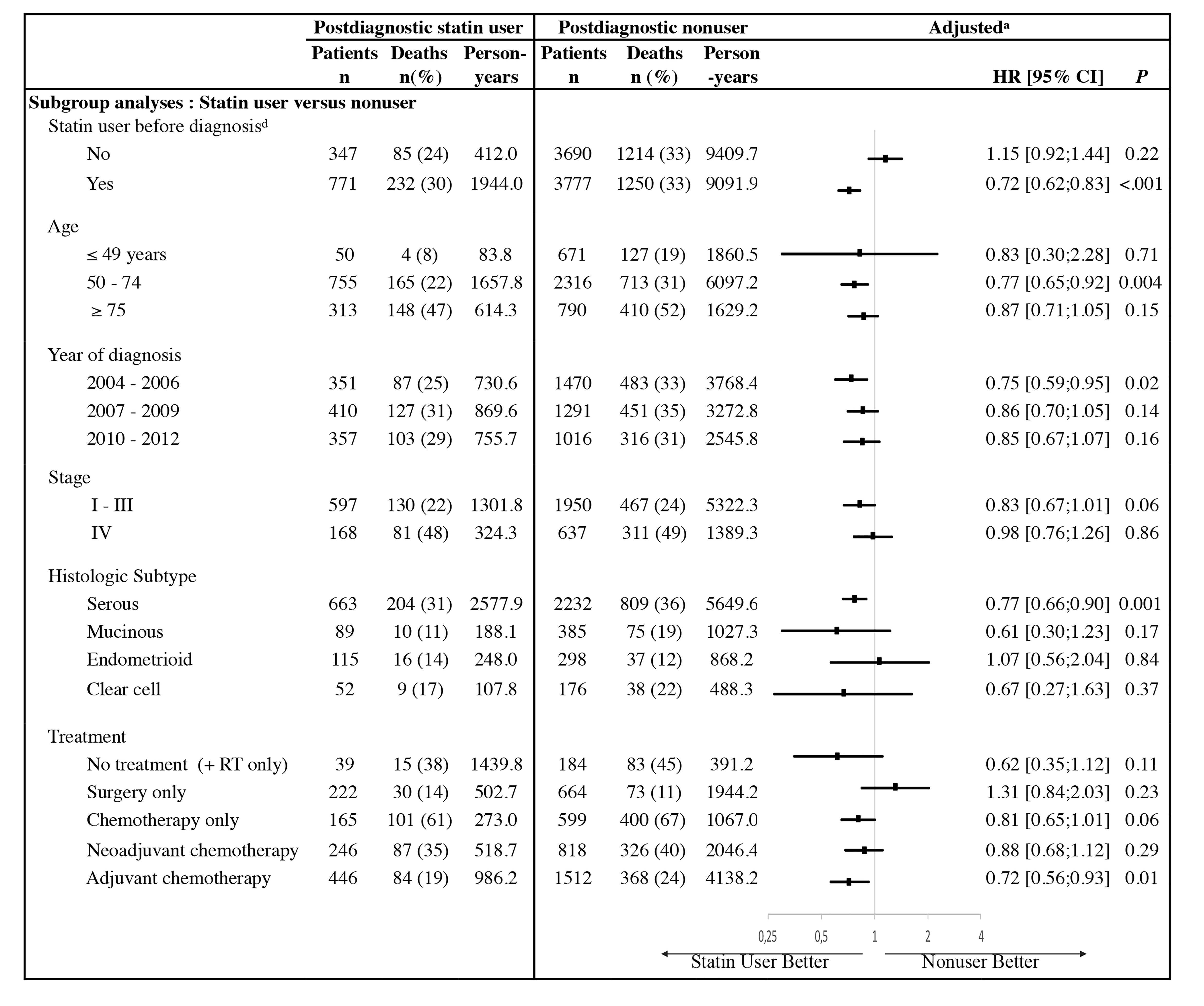

Supplement: S1 Fig — * CI denotes confidence interval. a Adjusted model contains age in categories (≤49 years, 50–74 years, ≥75 years), year of diagnosis (in 3-years bands), stage, cancer treatment within the 9 months (none, surgery only, chemotherapy only, neoadjuvant and adjuvant chemotherapy), comorbidities (diabetes and cardiovascular diseases). b Statin use before diagnosis was defined as at least one statin prescription in the year prior to the diagnosis. (TIF) [file pone.0189233.s003.tif]
